# Evaluating the Effectiveness of 3D‐Printed Models in Tooth Morphology Training for Students

**DOI:** 10.1002/jdd.70182

**Published:** 2026-02-21

**Authors:** Ivet Dzhondrova, Ilia Liondev, Hristina Galeva, Todor Bogdanov, Todor Uzunov, Dimitar Kirov

**Affiliations:** ^1^ Department of Prosthetic Dental Medicine, Faculty of Dental Medicine Medical University Sofia Bulgaria; ^2^ Department of Physics and Biophysics Medical University Sofia Bulgaria

**Keywords:** 3D‐printed teeth, 3D printing, dental education, dental models, tooth morphology

## Abstract

**Purpose/Objectives:**

Tooth morphology is a fundamental aspect of dental education, essential for various clinical disciplines. Traditional teaching methods, such as 2D visualizations, gypsum models, and wax or soap carving, have been widely used to develop students’ spatial understanding and manual skills. However, the integration of 3D‐printed models offers a novel approach that may enhance learning by providing a more accurate representation of dental morphology. This study aimed to assess the effectiveness of 3D‐printed models in learning tooth morphology by comparing their impact on students' practical and theoretical performance with traditional teaching methods.

**Methods:**

Dental students were divided into two groups: one utilizing 3D‐printed models alongside traditional methods and another relying solely on conventional gypsum and 2D visualizations. Practical assessments included soap tooth carving and wax modelling, while theoretical knowledge was evaluated through a written examination. Additionally, students completed a questionnaire to provide feedback on their experience with the 3D‐printed models.

**Results:**

Students who used 3D‐printed models achieved significantly higher scores in the soap tooth carving task, demonstrating improved accuracy and anatomical detail. However, there was no significant difference in the wax modelling scores between the two groups, suggesting that advanced manual dexterity skills are not solely dependent on 3D visualization. Theoretical examination results also showed no significant difference, indicating that 3D models primarily enhance practical skills rather than theoretical comprehension. Over 80% of students reported that 3D‐printed models were highly beneficial in understanding tooth anatomy and improving carving skills.

**Conclusion(s):**

The findings suggest that 3D‐printed models serve as a valuable tool for enhancing practical skills in dental education. While they do not replace traditional methods, they effectively support hands‐on learning and visualization.

## 1 Introduction

In the field of dentistry, the study of tooth morphology is a fundamental course, focusing on the composition, classification, and anatomical characteristics of individual teeth. The education of first‐year dental students typically begins with two‐dimensional visualization of teeth by drawing them from multiple perspectives. This practice helps students understand the basic anatomical differences between various types of teeth. As they progress, they develop their carving skills and enhance their ability to visualize and understand the dental structures in three dimensions. However, teaching tooth morphology has always presented challenges [1, 2]. Various innovative teaching methods have been adopted by dental schools to enhance learning outcomes [3, 4, 5, 6]. Despite these advancements, traditional textbooks using 2D images struggle to accurately represent the three‐dimensional (3D) morphological characteristics of tooth structures.

Over the past decade, Additive Technologies (AT) have developed at an exceptional pace, enabling the creation of complex and highly precise objects used in various fields, including dental medicine. 3D printing constructs physical models by layering materials based on digital design files (STL files), and the first to present it to the world was Charles Hull in 1986. This innovative technology enhances idea reproduction and has found applications across various disciplines [7]. In the field of dentistry, this technology has significantly contributed to advancements in clinical, experimental, and educational domains. Clinically, 3D printing enables the production of customized dentures, maxillofacial prostheses, crowns, bridges, orthodontic appliances, surgical guides, implants, and even 3D‐printed teeth [8]. Beyond clinical applications, 3D‐printed teeth have also been integrated into dental education [9, 10, 11]. Studies confirm that using these models enhances students' engagement and improves their preclinical skills [12, 13, 14, 15, 16]. Compared to prefabricated resin models (e.g., Frasaco or Nissin), which are widely used in dental education because they provide standardized morphology and relatively high durability, 3D‐printed models offer notable advantages. Prefabricated models provide standardized shapes but cannot easily be customized or scaled. In contrast, 3D‐printed models allow unlimited digital design modifications, enabling enlargement and reproduction of specific anatomical details [17, 18]. At the same time, 3D printing is more cost‐effective in the long term, as a single digital design can be repeatedly reproduced without purchasing new prefabricated sets [15]. This study evaluated the effectiveness of 3D printing technology in teaching tooth morphology by incorporating 3D‐printed tooth and jaw models aligned with traditional textbooks. Additionally, a questionnaire survey was conducted to assess students' perceptions of using these 3D‐printed models.

## 2 Methods

Study procedures were approved by the Institutional Review Board of the school.

### 2.1 Preparation of the 3D‐Printed Model Teeth

The preparation of the 3D‐printed model teeth was carried out prior to the beginning of the course to ensure standardized teaching materials for all students. First, the forth soap teeth were carved as models and scanned in a model scanner (3Shape F8, Copenhagen, Denmark) to obtain the data for the tooth printing (Figure 1). The printer, which was used for printing the resin model teeth, was Phrozen Mighty 8K (Phrozen Tech Co., Ltd., Hsinchu City, Taiwan) and features a 10‐inch LCD screen and operates at a 405 nm wavelength for effective polymerization. The model production was carried out using Anycubic Basic resin (Shenzhen Anycubic Technology Co., Ltd., Shenzhen, Guangdong, People's Republic of China), enhanced with CMYK Pigments from FEPshop (FEPshop Ltd., Groningen, Netherlands) to enable color customization. This resin‐pigment combination facilitated the accurate fabrication of detailed dental models suitable for clinical use.

**FIGURE 1:**
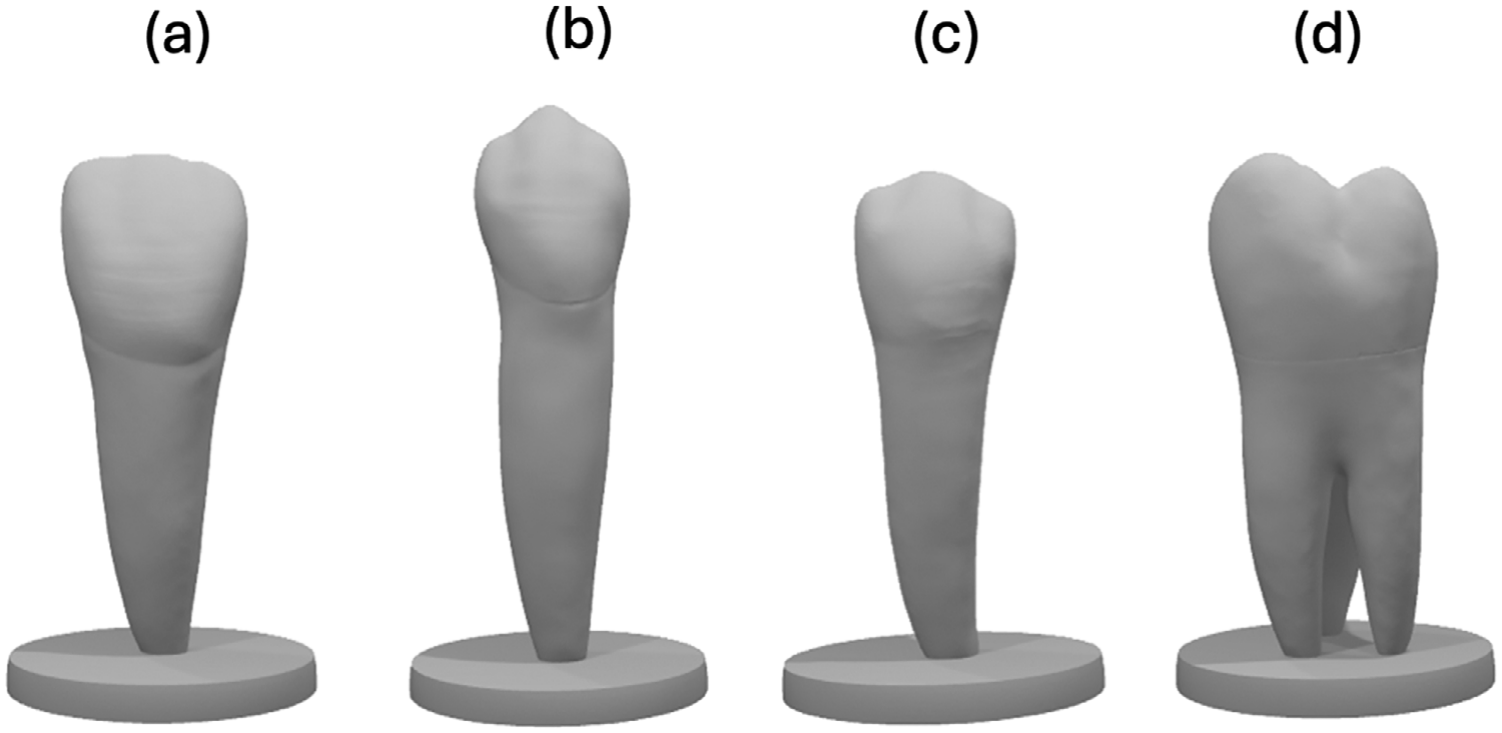
STL files of the model teeth: (a) upper central incisor, (b) upper canine, (c) lower first premolar, and (d) upper first molar.

### 2.2 Education Process

At this dental school, all dental students take courses on tooth morphology in their first year. Students begin their training by drawing all teeth from vestibular, lingual, medial, and incisal/occlusal views at a 3:1 scale. Once this step is complete, they proceed to carve teeth from soap, including a central incisor, canine, premolar, and molar. To successfully accomplish this task, students rely on their previously made drawings. The final task for students is to create five crowns using the wax modelling Thomas technique on two‐layered gypsum models with removable dies. Throughout the entire course, in addition to practical exercises, students also had theoretical sessions where the anatomical characteristics of each tooth were discussed.

### 2.3 Study Group

The study included 100 undergraduate students who enrolled in 2022 and 100 students who enrolled in 2024. The sample size was determined by the total number of first‐year dental students enrolled in the respective academic years (2022 and 2024). All participants engaged in the same teaching and assessment methods.

The control group (2022 students) and the experimental group (2024 students) were matched by year of study and curriculum structure. Baseline competencies were assumed to be comparable, as both groups had received identical preclinical training prior to the study. While the time span between cohorts introduces a potential source of variability, no changes in teaching staff, curriculum, or evaluation methods occurred during this period.

Both student groups were taught by the same instructors for the theoretical course and had the same practical instructors for soap tooth carving, with identical demonstrations presented. The theoretical examination administered to both cohorts consisted of the same set of questions, prepared by the same instructor and covering identical learning objectives. The structure, content, and level of difficulty were therefore consistent across both groups, ensuring comparability of theoretical assessment results.

Before the start of the course, each student enrolled in 2024 received a set of 3D‐printed plastic model teeth (central incisor, canine, premolar, and molar) (Figure 2) and used them during their practical exercises, whereas students who enrolled in 2022 relied solely on 2D visualizations, using their drawings. For the wax modelling task, students enrolled in 2022 worked on two‐layered gypsum models with removable dies, whereas those enrolled in 2024 used 3D‐printed models with removable 3D‐printed dies (Figure 3).

**FIGURE 2:**
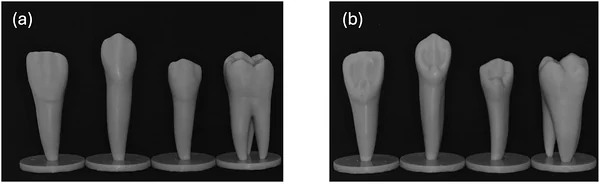
3D printed model teeth: (a) vestibular surface and (b) lingual surface.

**FIGURE 3:**
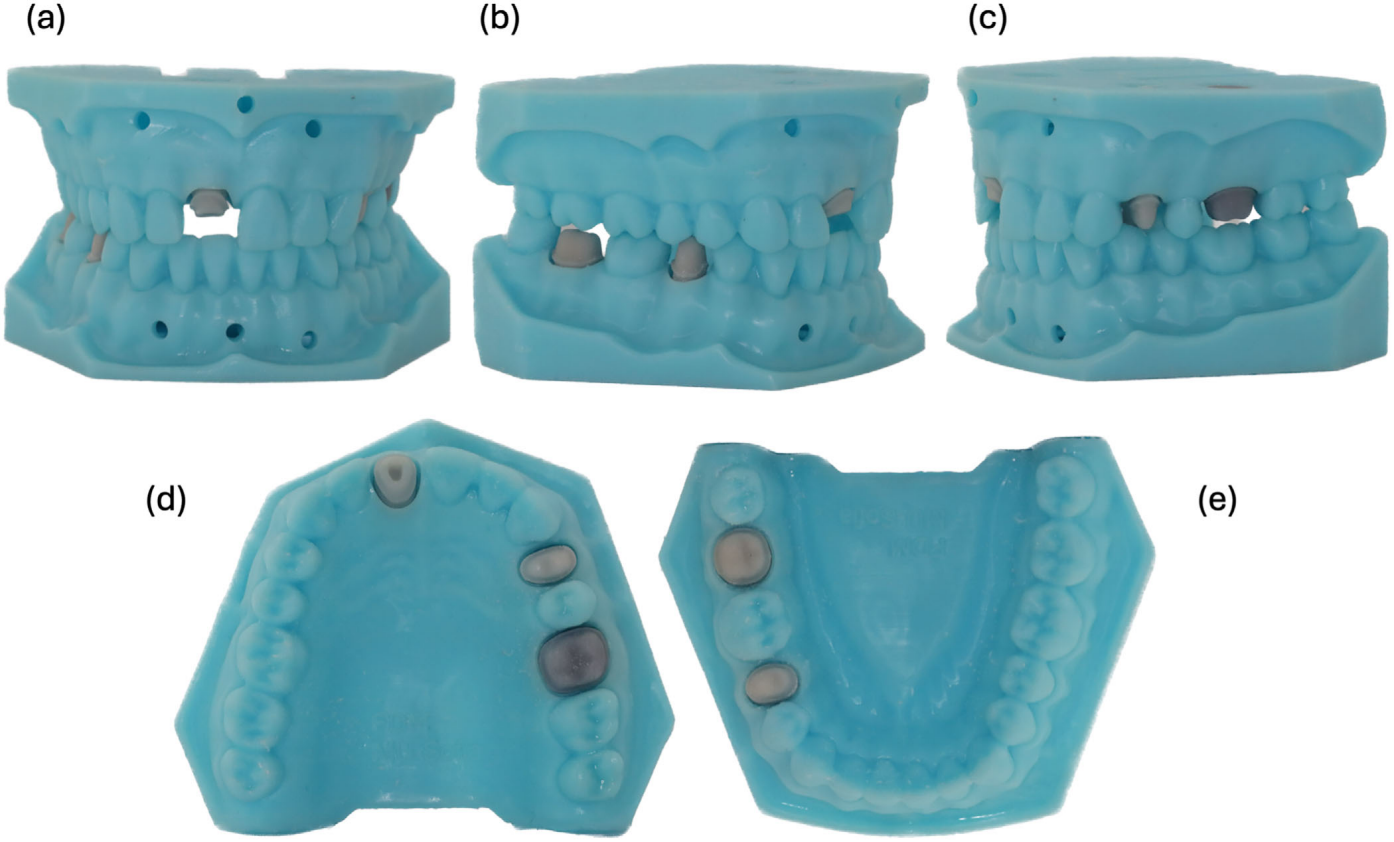
3D printed models for the wax modelling task: (a) frontal view, (b) left view, (c) right view, (d) upper jaw, and (e) lower jaw.

3D‐printed plastic model teeth and 3D‐printed models with removable dies were made from the same type of resin. This material was chosen because of its ability to present dental morphology in more detail, both in enlarged models and in those in real size. Also, the material is economically advantageous, as it takes about 1L of resin to make 8 sets of 3D printed models with five removable dies per set.

The enlarged 3D‐printed teeth models provide all students with unified 3D examples of the expected final result. They help to better visualize the tooth morphology, but also to orient students easier when learning the anatomical features of the different tooth surfaces for each individual group of teeth.

Working on 3D printed models helps to reduce and even avoid the presence of inaccuracies and artifacts in working models (such as air bubbles, pearls, or unclear occlusal relief). The hardness and mechanical properties of the material make it more resistant than gypsum during work—both in the thermal changes that occur (part of the teeth modeling technology/, and in the case of the need for multiple destruction and repetition of various elements of the modelling.

Due to their sustainability, this type of model can also be used by other students in their next course of study. The dusting of the fine gypsum powder during the making of the plaster models and working with them is eliminated. This is good for the health of everyone in the room and makes working with these models significantly cleaner.

At the end of the course, students completed an anonymous questionnaire to evaluate the effectiveness of the 3D‐printed models in enhancing their learning experience.

### 2.4 Examination

The theoretical assessment is conducted through a written exam, focusing on the anatomical and morphological characteristics of each tooth, as well as comparisons between different teeth. The exam includes various question types, primarily multiple‐choice questions. The total score for the assessment is 12 points, with scores of 12 and 11 considered excellent, 10 and 9 very good, 8 and 7 good, 6 and 5 average, and below 5 classified as a low grade.

After the theoretical assessment, students are required to hand in a set of carved soap teeth. The score is based on the accuracy of the shape and the precision of the detailed carving in the soap teeth. The grade of each tooth is as follows: 6 points—excellent for correct shape, size, and details, 5 points—very good for correct shape and size without details, 4 points—good for correct shape with incorrect size and without details, 3 points—average for incorrect shape, size, and details.

Finally, students are required to present five wax tooth crowns. The grading criteria for each tooth are as follows: 6 points—excellent for correct shape, size, and details; 5 points—very good for correct shape and size but lacking details; 4 points—good for correct shape but incorrect size and missing details; 3 points—average for incorrect shape, size, and details.

The total score of the practical and theoretical examination is 24.

Each student's work was independently assessed by two calibrated instructors using standardized criteria, including anatomical accuracy, proportional symmetry, surface smoothness, and margin integrity. To ensure consistency, calibration sessions were conducted prior to assessments, where instructors reviewed sample carvings. If the score difference between the two assessors was ≤ 2 points, the average was recorded. If the difference exceeded 2 points, a third calibrated instructor was invited to reassess the work. The final score was determined by averaging the two closest evaluations. The third instructor was randomly selected from the pool of evaluators, ensuring impartiality. Both practical tasks (soap carving and wax modelling) were evaluated by the same group of calibrated instructors in both academic years.

For the soap carvings, the corresponding 3D‐printed model served as the reference standard to ensure comparability across students. All soap carvings were graded individually, and the final score was calculated as the arithmetic mean of the evaluations. For the wax crowns, each of the five crowns was assessed with equal weight, and the overall score represented the arithmetic mean of the five grades.

### 2.5 Statistics

IBM SPSS Statistics for Windows, Version 24 (IBM Corp., Armonk, New York) was used for statistical analysis of the data. The scores of the two groups of students in the theoretical assessment and soap carving, and the total scores, were in line with the normal distribution and homogeneity of variance. The scores of the two groups of students in the theoretical assessment and soap tooth carving, as well as the total scores, were tested by a *t*‐test, and *p* < 0.05 was considered statistically significant.

## 3 Results

### 3.1 Comparison of Scores Between the Two Groups of Students

There was no significant difference in theoretical examination scores and total scores between the two student groups. However, students who enrolled in 2024 and used 3D‐printed plastic model teeth in their classes achieved significantly higher scores on their carved soap teeth compared to those who did not use the models (Table 1).

**TABLE 1:** Comparison of scores between the two groups of students.

Class	Theoretical examination score	Tooth‐carving score	Wax modelling score	Total score
Students enrolled in 2022 (100)	8.62 ± 2.35	3.97 ± 0.83	4.59 ± 0.83	17.18 ± 3.11
Students enrolled in 2024 (100)	8.21 ± 1.83	4.58 ± 0.97	4.64 ± 0.87	17.43 ± 2.58
	(*p* = 0.08)	(*p *< 0.00001)^*^	(*p* = 0.34)	(*p* = 0.61)

* The result is significant at *p* < 0.05.

### 3.2 Survey Results of Students Who Matriculated in 2024

Table 2 presents the results from the feedback of students enrolled in 2024 who worked with 3D‐printed models, and 70% of them agreed that 3D‐printed tooth models are helpful when carving teeth from soap bars, with 39% finding them absolutely helpful. In contrast, only 16% of students believed that the traditional tooth drawings clearly represented all anatomical features and were sufficient for learning dental morphology, while more than half (52%) felt they were not sufficient. A large majority (89%) indicated that working with 3D‐printed models made it easier to understand tooth morphology, with 51% responding “absolutely” and 38% “mostly.” Furthermore, 72% of students preferred 3D‐printed models over traditional plaster ones when performing wax modelling. Regarding ease of use, 48% of students found 3D‐printed models easier to work with, while 45% rated them as having similar difficulty to plaster models. When asked if 3D‐printed models should completely replace plaster models in dental education, the majority (61%) preferred a combination of both approaches. As for the perceived advantages of 3D‐printed models, 30.3% appreciated their use as references during carving or wax modelling, 28.1% valued the greater detail compared to traditional drawings as a 2D visualization method, and 22.5% noted better visualization of dental morphology. These findings highlight the potential of 3D‐printed models to enhance the teaching of dental anatomy by offering improved clarity, usability, and practical application compared to traditional 2D or plaster‐based educational tools.

**TABLE 2:** Results of the feedback questionnaire for students who matriculated in 2024.

**Question**	**Options**			
(1) Do you think the 3D‐printed teeth models can be helpful when carving teeth out of soap bars?	Absolutely (39%)	Mostly (31%)	Somewhat (16%)	Not at all (14%)
(2) Do you think the tooth drawings you used for carving clearly represent all anatomical features of the teeth and are sufficient for learning dental morphology?	Absolutely (16%)	Mostly (12%)	Somewhat (20%)	Not at all (52%)
(3) Do you think working with 3D‐printed models makes it easier to understand dental morphology?	Absolutely (51%)	Mostly (38%)	Somewhat (8%)	Not at all (3%)
(4) Do you prefer 3D‐printed models over traditional plaster models when working with wax modelling?	Absolutely (42%)	Mostly (30%)	Somewhat (17%)	Not at all (11%)
(5) Compared to plaster models, 3D‐printed models are:	Easier to work with (48%)	The same in terms of difficulty (45%)	More difficult to work with (7%)	
(6) Do you think 3D‐printed models should completely replace plaster models in dental education?	Yes (28%)	No (11%)	A combination of both methods would be best (61%)	
(7) What do you think are the advantages of a 3D‐printed dental models? (multiple choice)	Better visualization of dental morphology (22.5%)	Show more details compared to drawings (28.1%)	Can be used as a reference when carving or wax modelling (30.3%)	Easier to compare anatomical features of different teeth (17.4%)

## 4 Discussion

Tooth morphology is a fundamental aspect of dental education, forming the basis for various clinical disciplines such as restorative dentistry, prosthodontics, and orthodontics. A thorough understanding of anatomical characteristics is essential for diagnosing dental conditions, designing restorations, and performing precise clinical procedures. Traditional teaching methods, such as 2D drawings, soap or wax carving, and wax modelling, have long been used to develop spatial awareness and fine motor skills. However, integrating modern educational tools, such as 3D‐printed models, may offer additional benefits by providing a more accurate and detailed representation of dental structures, as demonstrated in previous studies [3, 9, 10, 11].

The results of our study demonstrated that students who used 3D‐printed models performed significantly better in soap tooth carving tasks compared to those relying solely on traditional 2D visualization methods. This suggests that the enhanced spatial representation provided by the 3D models contributed to a more precise understanding of tooth morphology, allowing students to apply this knowledge more effectively in practical exercises. Students also reported that 3D models improved their ability to visualize dental structures, making practical work more intuitive and engaging. These findings align with research by de Boer et al. [16], which showed that 3D visualization enhances student performance and engagement in virtual learning environments, reinforcing the idea that 3D learning tools significantly improve practical skills in dental education.

However, when evaluating the grades for wax modelling, no significant difference was observed between students who worked on 3D‐printed models with removable dies and those who used two‐layered gypsum models. This suggests that while 3D models may enhance early learning stages, such as visualization and soap carving, they do not necessarily translate into improved performance in more advanced manual tasks like wax modelling. One possible explanation is that the motor skills required for wax modelling are developed through repeated practice rather than solely through enhanced visualization, highlighting the continued relevance of traditional techniques for refining these skills.

The results of the written examination showed no significant difference between the two groups, indicating that the use of 3D‐printed models did not impact students' theoretical knowledge of tooth morphology. Since both groups received the same theoretical instruction, this suggests that 3D models primarily enhance practical skills rather than theoretical comprehension. Similarly, studies by Wang et al. [12] and Alzahrani et al. [19] found that while innovative teaching methods improved carving skills, they did not significantly influence theoretical knowledge, reinforcing the idea that theoretical assessments primarily test memorization rather than hands‐on experience. This underscores the importance of integrating both traditional and modern teaching methods to ensure a well‐rounded dental education. While 3D‐printed models provide valuable support for visualization and skill development, they should serve as a complement rather than a replacement for conventional theoretical learning approaches. Importantly, within the context of tooth morphology education, the primary learning objectives extend beyond factual knowledge to encompass spatial comprehension, 3D visualization, and the development of fine motor skills. These competencies are fundamental for subsequent clinical training but are not fully reflected in traditional written examinations.

The results from the student questionnaire further support the effectiveness of 3D‐printed models. A majority of students reported that these models improved their ability to visualize tooth structures more accurately and facilitated a deeper understanding of anatomical features. Many also indicated that working with 3D models made their practical exercises easier and more intuitive. Research conducted on students' interest in implementing digital technologies, including printed 3D working models in dental education, confirms its positive effect. When comparing the work on plaster models and the work on 3D printed models, students clearly show a preference for the printed ones [20].

Another notable advantage of 3D‐printed models is their durability and reproducibility. Unlike gypsum models, which are fragile and prone to breakage, 3D‐printed models can withstand repeated handling and can be easily reproduced if lost or damaged. Additionally, they allow for a standardized learning experience, as every student receives an identical model, eliminating inconsistencies that may arise with hand‐made gypsum models. However, Bogdanov et al. find that mechanical handling plays a significant role in model quality. Damage analysis shows a correlation between the mechanical resistance of the printed models and the change of forces and temperatures during work [20].

While the benefits of 3D‐printed models in enhancing visualization and skill development are clear, certain limitations must be addressed. The production of 3D‐printed models requires specialized printers, materials, and software, which can be costly for some institutions, potentially limiting widespread adoption, particularly in resource‐limited settings. Additionally, most 3D‐printed models are made from non‐biodegradable plastics or resins, raising concerns about environmental impact. Unlike gypsum, which is more easily recyclable, current 3D printing materials have limited disposal and recycling options.

A study by Mahrous et al. [3] assessing student perceptions of various learning tools ‐including natural teeth, 3D‐printed models, 3D virtual models, and augmented reality (AR) technology—found that each method had distinct advantages. Natural teeth were rated highest in educational value, while 3D‐printed models were considered the most accessible and easiest to use. These findings align with our study, which demonstrated that 3D‐printed models enhance practical skills in dental morphology education. The accessibility and ease of use of 3D‐printed models make them a valuable tool for preclinical dental training. However, as noted in Mahrous et al.’s study, drawbacks such as the cost of 3D printing technology and materials remain a consideration.

A limitation of this study is the use of two separate academic cohorts as control and experimental groups, which may introduce cohort‐related variability that cannot be fully excluded. Nevertheless, both cohorts were enrolled within the same curriculum framework, received identical theoretical instruction, and were assessed using standardized and identical examination materials, helping to ensure comparability between groups. Another limitation is the absence of a pre‐course assessment, which prevented formal verification of baseline equivalence in theoretical knowledge and manual skills. However, all participants were first‐year dental students with no prior formal training in dental technology or preclinical dental procedures, thereby reducing variability in baseline experience. Additionally, because multiple 3D‐printed teaching aids were introduced simultaneously, the specific contribution of each component could not be independently determined. Future studies should incorporate pre‐ and post‐testing designs and, where feasible, randomized group allocation to more precisely isolate the educational impact of 3D‐printed models.

Our research highlights the valuable role of 3D‐printed models in enhancing student learning by improving visualization, accuracy, and engagement. While these models should not completely replace traditional teaching methods, they can serve as an effective supplement to existing educational tools. Future research should further assess the long‐term impact of 3D‐printed models on clinical skill development and explore the potential of advanced educational tools such as 3D visualization and augmented reality to enhance dental training.

## 5 Conclusion

This study examined the impact of 3D‐printed models on dental education, particularly in learning tooth morphology. The results indicate that while 3D‐printed models significantly improve students’ practical skills, especially in carving accuracy and detail, they do not influence theoretical knowledge, as exam scores remained similar between groups. Students who used 3D‐printed models achieved higher scores in carving soap teeth, and over 80% found them highly beneficial for understanding tooth anatomy and enhancing carving skills. By transforming textbook illustrations and drawings into 3D structures, these models effectively support hands‐on learning and skill development, making them a valuable addition to traditional teaching methods.
